# ECOA-9. Integration of molecular and methylation classifications yields three meningioma groups and suggests chromosome 1p loss may be critical to the aggressive group

**DOI:** 10.1093/noajnl/vdab070.009

**Published:** 2021-07-05

**Authors:** James Bayley, Caroline Hadley, Arif Harmanci, Akdes Harmanci, Tiemo Klisch, Akash Patel

**Affiliations:** 1 Baylor College of Medicine, Houston, TX, USA; 2 University of Texas Health Sciences Center at Houston, Houston, TX, USA

## Abstract

Next-generation sequencing has deepened our understanding of meningiomas, particularly those that behave aggressively. Classifications using either DNA methylation profiling or RNA-sequencing predict tumor behavior more reliably than WHO grade, and segregate common meningioma features similarly, implying possible overlap between classifications. In this study, we performed DNA methylation profiling, RNA-sequencing, and whole-exome sequencing on 110 primary meningiomas (90 WHO I, 20 WHO II). Unsupervised non-negative matrix factorization demonstrated three epigenetic types which were highly concordant with our published transcriptional types (87.3% concordance). Two additional classifications (one using 1p/22 loss, the other merlin expression/chromosomal instability) were also highly concordant and an overall meningioma group (MenG) classification was assigned integrating all four together. MenG A and B rarely recur, while MenG C behave aggressively (median recurrence free survival (RFS) of 3.1 years), even after gross total resection (median RFS 4.2 years). MenG A tumors retain Merlin expression (no chromosome loss or NF2 mutation) and harbor mutations in TRAF7, AKT1, or KLF4. Both MenG B and C are merlin-deficient, but MenG B demonstrate low rates of CNV and MenG C high rates of CNV, particularly loss of chromosome 1p. Using partial least squares regression (PLS), we explored how gene expression correlated with promoter methylation and CNV, thereby classifying genes which correlated closely as ‘methylation-driven’ or ‘CNV-driven’. Overall, there were more methylation-driven (5.7%) than CNV-driven (2.9%) genes. Differentially expressed genes (DEGs) were enriched for both methylation- and CNV-driven genes at similar proportions (10.6% and 5.8%), but DEGs unique to MenG C were significantly enriched for CNV-driven (23.7%), but not methylation-driven (4.7%) genes, primarily due many MenG C DEGs on chromosome 1p. Overall, this work suggests three underlying meningioma groups which are identifiable through methylation, transcriptional, or genetic/cytogenetic profiling and warrants exploration of the role of chromosome 1p in group that behaves aggressively.

